# Quality control on oil palm RNA samples for efficient genomic downstream applications

**DOI:** 10.7150/jgen.92209

**Published:** 2024-02-17

**Authors:** Ardha Apryianto, Primadiyanti Arsela, Foncha Felix, Walter Ajambang

**Affiliations:** 1 Institute of Agricultural Research for Development (IRAD), Cameroon.; 2 Biopolymer Analytics, Institute of Biochemistry and Biology, University of Potsdam, Karl- liebknecht-str. 24-25 Potsdam Golm- Germany.; 3 Laboratprium Bioteknologi, ASTRA Agro Lestari Ltd Indonesia.; 4 Prodi Agroteknologi, Fakultas Pertanian dan bisnis Digital, Universitas Muhammadiyah Kalimantan Timur, Indonesia.

**Keywords:** OD, RIN, RNA, genomics, oil palm

## Abstract

Quality control (QC) is primordial for determining the efficiency in any downstream genomic applications. There are several steps in the verification of the quality of RNA samples destined for genomic studies. The aim of this research was to determine whether RNA should be discarded at the level of the field lab if it fails preliminary quality control using Optical Density (OD) measurements. In this study, all samples were submitted to rigorous quality control in every stage of work. RNA samples showing poor OD values still gave excellent results in downstream QC and genomic applications. At the end of the quality control exercise, it was observed that the original samples were the same and had not undergone any deterioration along the different stages of handling and manipulation. This paper shows the different and most important stages of quality control on RNA samples (RIN) for an effective down stream application in genomic studies. RNA samples should not be discarded based on preliminary QC from our field labs.

## Introduction

Oil palm is an important economic crop covering more than 15 million ha in the tropics and semi tropics. It contributes 37% of global vegetable oil production. Oil palm is undergoing vast expansions and replanting in all the major producing countries. It is estimated that oil palm has an expansion rate of 3.5% in Indonesia. The production of palm oil has been a major source for conflicts because of land and environmental concerns. Governments want to limit the expansion of oil palm cultivation because of climate and food security claims. It would be preferable to increase the productivity of the present land rather than trying to expand its total cultivation areas. One of the ways to improve productivity is through breeding for high production. Conventional breeding in oil palm takes more than 15 years to develop and release a single variety. Most researchers, who start breeding programs in oil palm institutions, go on retirement before their projects are completed. Most of the projects uncompleted are shelved after the principal investigator goes on retirement. Genomics have revolutionized breeding activities in all major crops including oil palm. RNA-seq through Next Generation Sequencing (NGS) has brought a revolution to genomic studies. RNA-seq is a high throughput technology that is widely used currently to elucidate the transcriptomic activities and other biological pathways in living organisms 1. Extracting RNA from plant tissues can be difficult especially when working on woody perennials such as the oil palm which contains high levels of extractable phenols and polysaccharides 2. Oil palm is rich in polysaccharides and polyphenols, with leaves having a waxy tissue and high fibre content 3, 4, 5. The chemical nature of RNA molecules, that is very susceptible to hydrolysis and sensitive to degradation by widespread, stable ribonucleasess, makes RNA isolation difficult 6 - 10.

Most Oil Palm Research Centres own laboratories that can barely boast of minimum equipment for pre-genomic studies. These field laboratories are used to prepare materials for complex down stream applications that would be handled by distant specialized institutions located in bigger cities or in more technologically advanced countries. The field laboratories need to be sure that the materials that they are sending for down stream genomic applications do meet the required standards, hence they have to undergo primary quality control in these field laboratories. Purification of high quality and high integrity RNA from plant samples is an important and crucial prerequisite for the success of down stream applications such as cDNA reverse transcription, hybridization studies, and NGS. Isolation, purification and handling of RNA are more delicate as compared to DNA due to the presence of ubiquitous and stable ribonuclease enzymes in plant tissues, which can rapidly degrade RNA 8, 9.

Nucleic acid purity is estimated by the ratio of absorbance contributed by the nucleic acid to the absorbance of the contaminants, usualy denoted as A_260_/A_280_. Absorbance at 260 nm measures the amount of nucleic acid present in the sample while absorbance at 280 nm measures the amount of protein and other aromatic amino acids in the sample 10 - 14.

OD ratios for RNA purity generally acceptable for downstream applications range between 1.8 and 2.2 10 - 14.

Although the A_260_/A_280_ ratio is appropriate for estimating nucleic acid purity, the amount of genomic DNA found in the RNA sample cannot be determined by absorbance. The absorbance value may also not be real if a significant amount of some contaminants absorbing wavelengths around 260 nm are found in the sample. In addition, RNA can degrade during the handling process for onward transmission to downstream operations. However, there are other methods that measures RNA integrity (RIN) and degradation, this by using the 2100 Bioanalyser.

Quality control for RNA-seq through NGS activities is carried out in three different stages. The first stage consists of measuring the concentration and optical density of RNA and DNA. This stage can be carried out in most field labs. The second stage that needs the use of a Bio-analyser can only be carried out in specialized laboratories located in far away cities. The third stage is the quality control of the sequenced data that is carried out farther from the plantations and usually in countries which have the required sequencing technology.

It would be interesting and even of prime importance if researchers can be able to predict the outcome of sequenced data quality by just looking at the results of RNA quality (OD measures) obtained in their field labs.

The following hypothesis drove us to conduct this research.

Our hypotheses were that;

1. That the tissues with good quality and high concentration (high OD) as measured at the field lab conditions, would further have higher RIN and rRNA ratio figures and vice versa.

2. Post-isolated RNA degradation is not tissue specific under similar handling processes.

3. Tissues from all the different organs of oil palm shall have the same quality and quantity when their RNAs were extracted using the same extraction kit.

### Objectives of this study

To determine whether RNA samples showing low quality in the field labs through OD measures, could still give good results in down stream genomic applications.

## Methods

**Plant organs and developmental stages used**: Four different tissues at various physiological levels of maturity were selected for RNA isolation. The 4 different tissues were; A1 = flowers between leaf number -22 and leaf number -28, A2 = flowers between leaf number -18 to leaf number -21, A3 = flower at the axil of leaf number +4 and A4 = young leaf tissues. The tissues are older as we move from stage 1 to 4. Five biological replicates were palm trees A, B, C, D and F. Details of these different samples are explained in 15.

**Tissue extraction**: Flowers located on adjacent leaf axils were grouped to make 100 mg whenever the required quantity was not attained. Otherwise, the tissue was reduced to meet the 100 mg requirement. The same was done for leaf tissues.

**RNA isolation**: Total RNA was isolated from the tissues using the RNeasy Plant Mini Kit (Qiagen®, Inc. Valencia, CA, USA) with slight modifications. In the absence of liquid nitrogen at the laboratory in Kalimantan, we slightly increased the quantity of the RLC® lysis buffer to 500 µl and the buffer was added directly to the tissue in the mortar prior to grinding. Vortexing was omitted and the samples were directly transferred into the QIAshredder® spin columns 15.

**RNA quantification and quantity**: The Nanodrop ND-2000 spectrophotometer (Thermo Fisher Scientific Inc, Waltham, MA, USA) was used to measure the concentration and optical density of the RNA samples. The optical density (OD) measured as a ratio of A260/A280 of all samples was between the required range of 1.8 and 2.2. RNA concentration was very high ranging between 300 ng/µl and 1600 ng/µl in 40 µl of total RNA volume 15.

**RNA stabilization and shipping**: Sequencing of the transcriptome was done in Korea by Macrogen®. Transporting the RNA from Kalimantan to Jakarta and then to Korea warranted some preservation because RNA rapidly degrades at room temperature. We used RNAstable® (Biomatrica) for the stabilization of the RNA. We collected 25 µl out of the total 40 µl and added into the tubes containing RNAstable. The tubes were carried to a vacuum drier and dried at 15 °C for 2 hours. The samples were collected in the tubes and stored in a heat-sealed moisture barrier aluminum foil bag containing 2 silica gel desiccant packets. The bag was placed in room temperature regulated at 20 °C for 1 week before shipment. The remaining 15 µl of RNA was stored under - 20 °C as a back up for the samples 15.

**RIN and rRNA ratio measurement**: This was done by measuring the characteristics of the electropherogram generated by an Agilent 2100 Bioanalyzer, including the fraction of the area in the region of 18S and 28S rRNA, the height of the 28S peak, the presence or absence of RNA degradation products, the fast area ratio and marker height. RIN values range from 1 for completely degraded samples to a value of 10 for completely intact RNA 15.

## Results and Discussion

### Optical Density values

Table 1 shows the total list of RNA samples and their accompanying codes. The total concentration of each sample is given in ng/µl. The different peak values from Nanodrop are also given. The values show that the quality of our RNA at the level of the lab is of very good quality. We see that irrespective of the RNA concentration, the OD standard purity value is attained. The values tie with those proposed by 14 on the purity of RNA based on OD.

### Further quality control and RIN Values

Table 2 shows RIN values from the different RNA samples. Although the corresponding quality control done in the field lab showed that the RNA was of very good quality, the corresponding RIN values show that, there are some discrepancies in the quality. RIN is measured in a scale of 1 to 10. The standard values for best quality RIN are between 7 and 8 18. Most samples have values oscillating between the standard RNA RIN value. Irrespective of OD values, samples behaved differently in the later stage of QC verification RIN.

### Comparing quality values from NANODROP against the quality from BIOANALYSER

**Figure 1:**
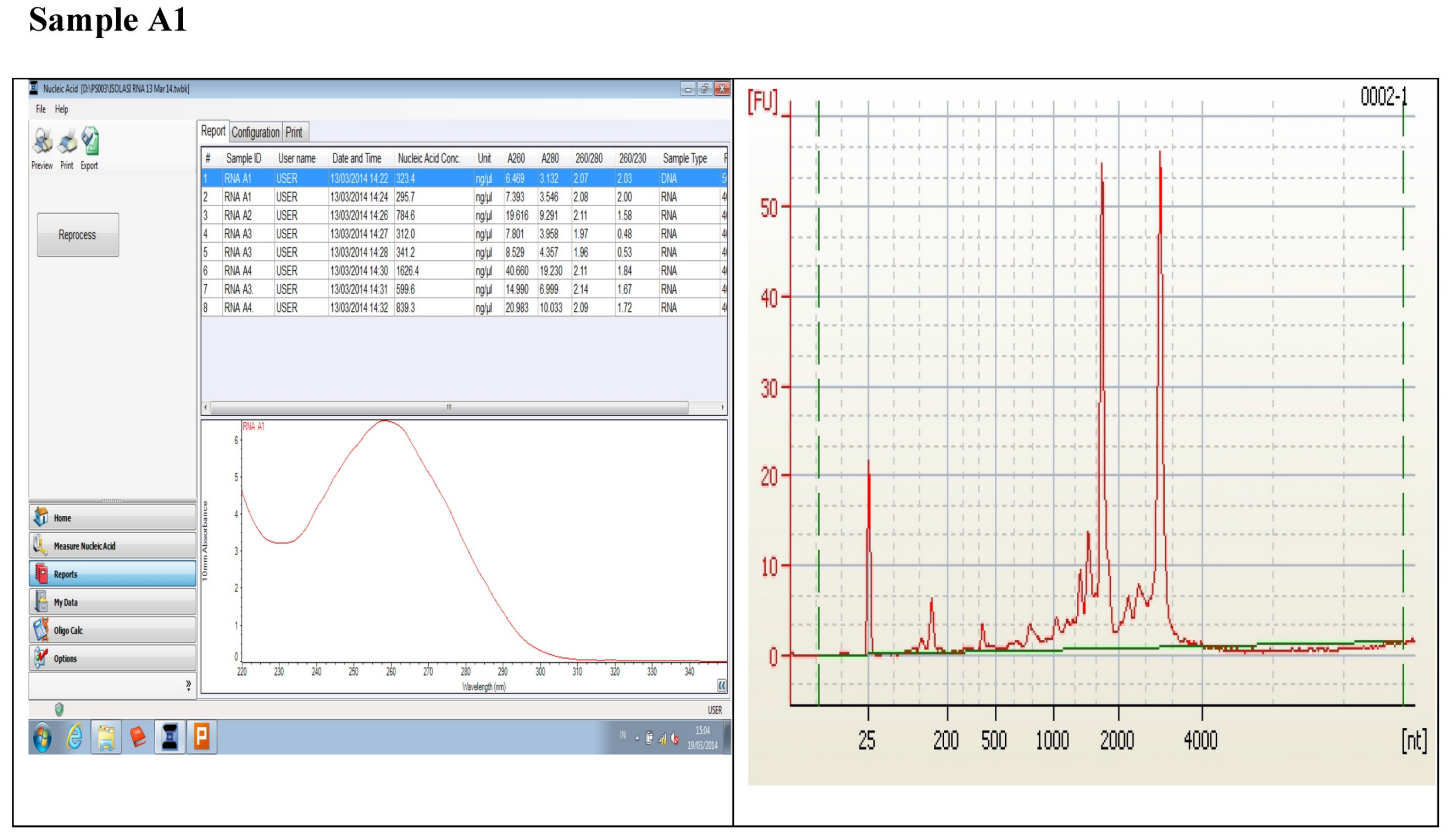

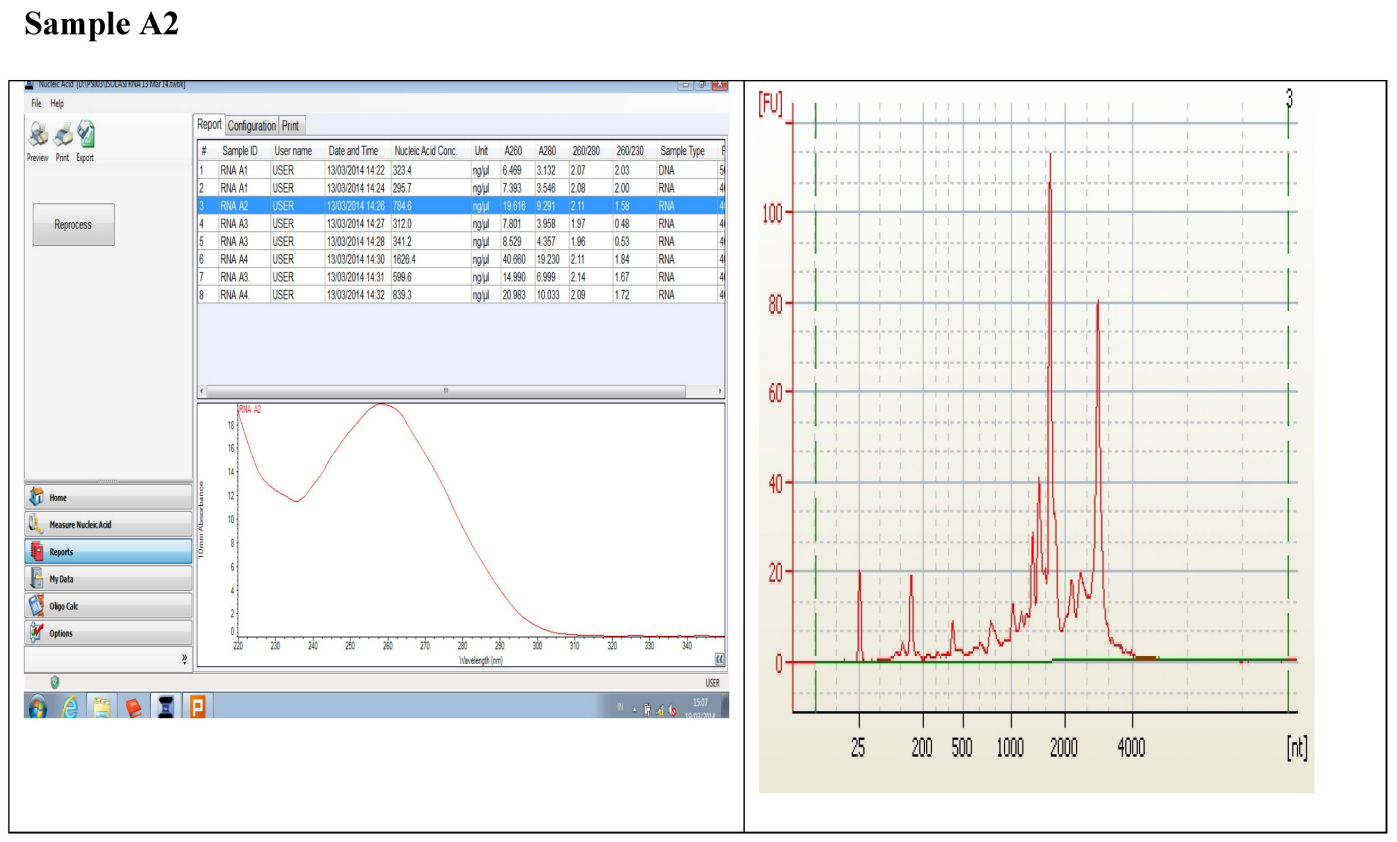

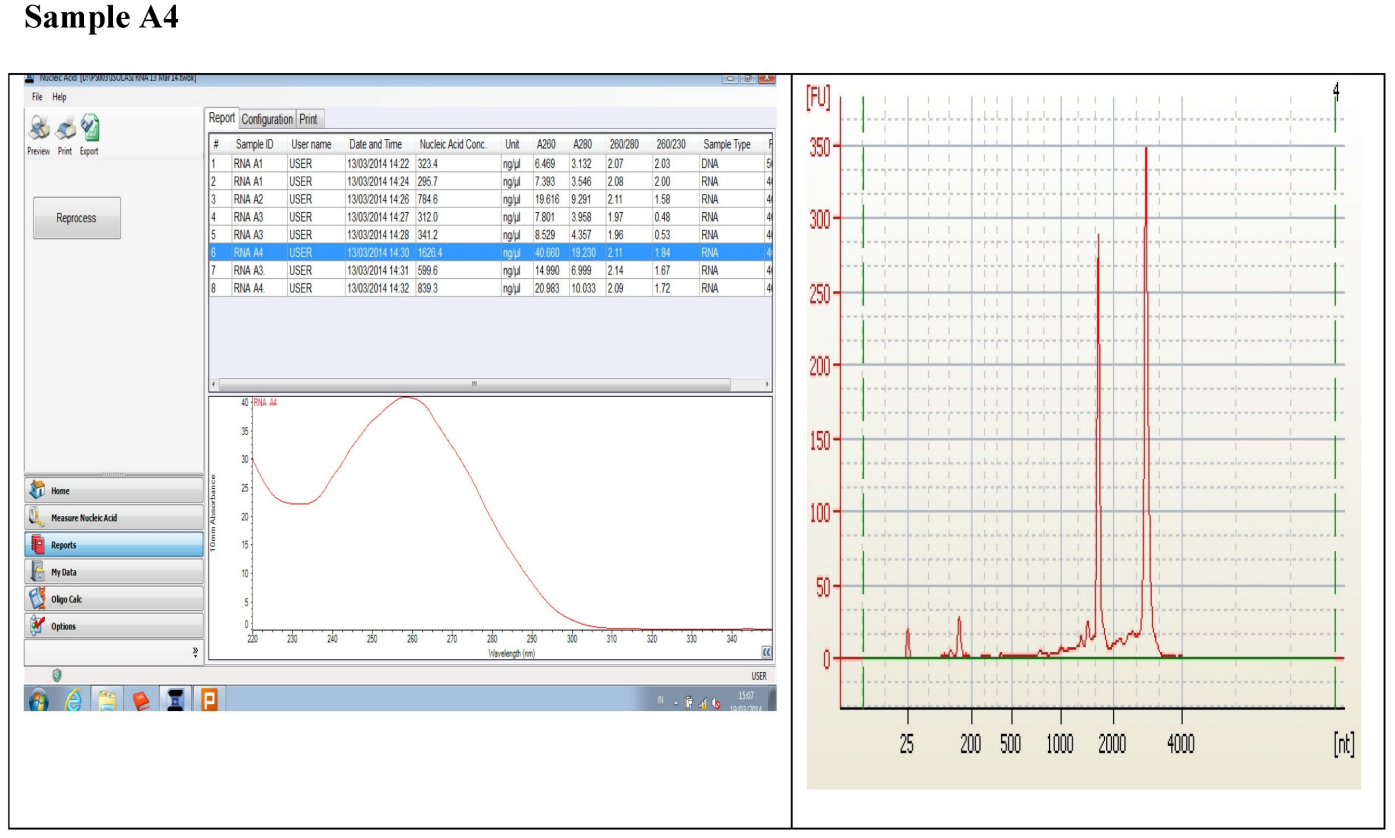

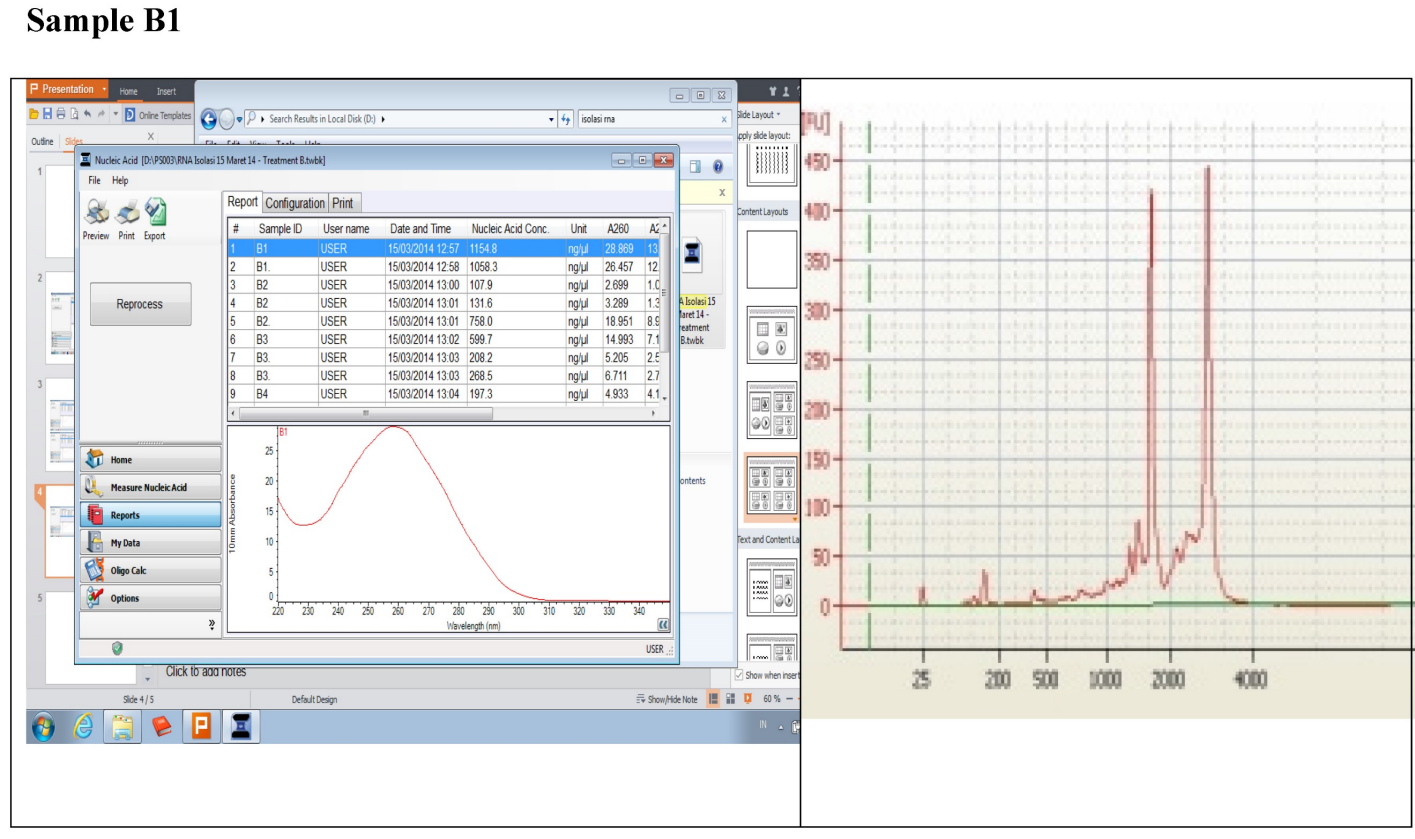

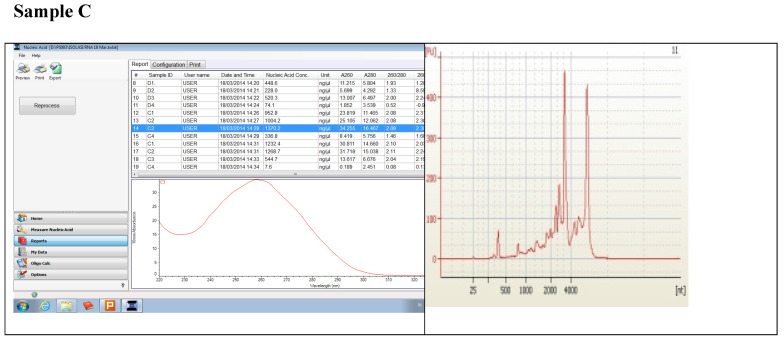

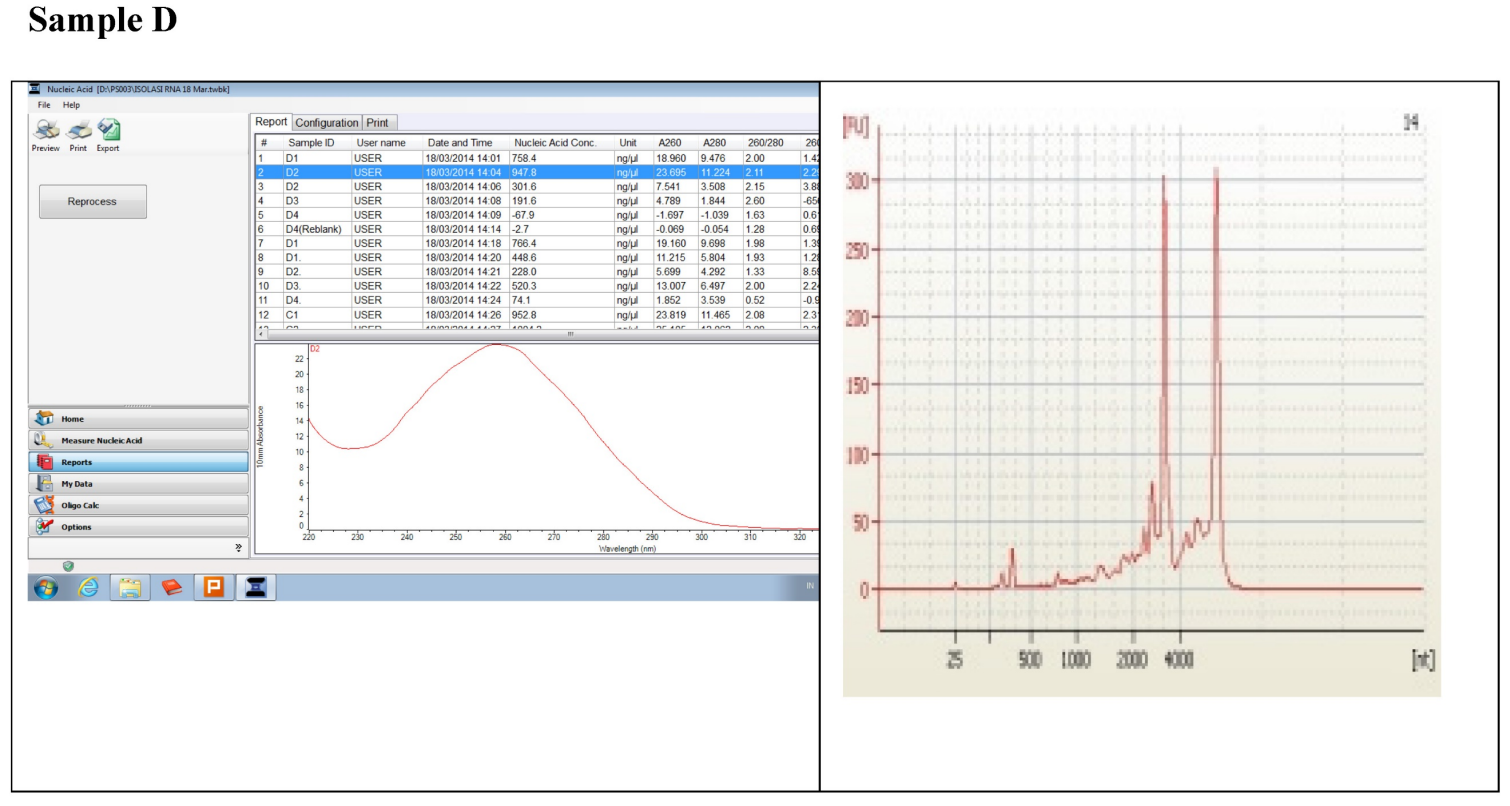

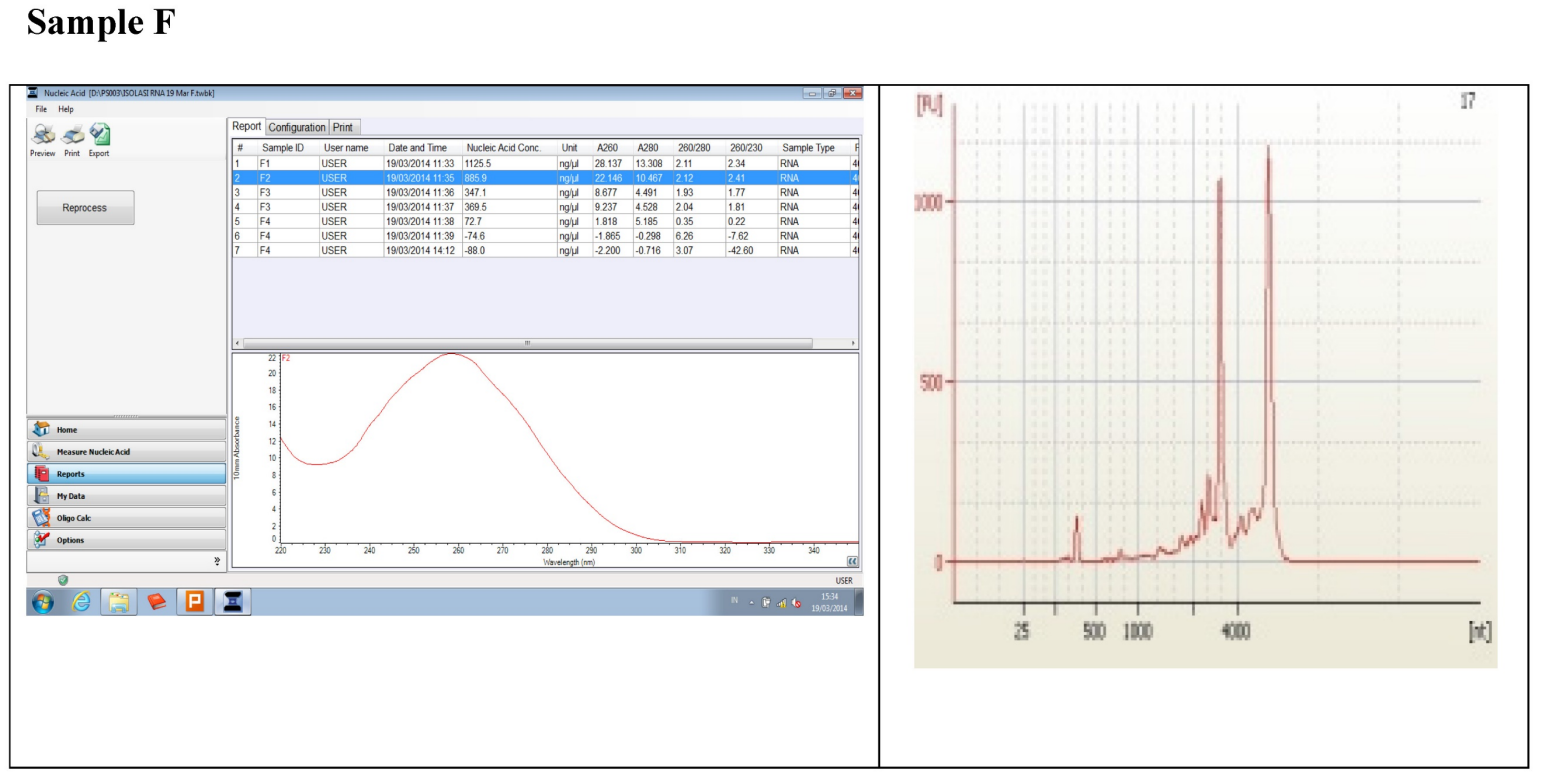
Comparing quality values from NANODROP against the quality from BIOANALYSER.

Figure 1 displays graphs of OD quality control at the level of the field laboratory alongside graphs of corresponding RIN values from Bioanalyser. We can observe that the best ODs do not usually yield the best RIN values. The OD and RIN should be measuring completely different properties of the same RNA sample. Both values are extremely important for the downstream genomic applications using these samples 16, 17. This is an indication that RNA samples presenting poor OD values measured in the field labs shouldn't be discarded immediately but be forwarded for RIN determination.

### Correlations between DNA concentration, rRNA ratio and RIN

**Figure 2:**
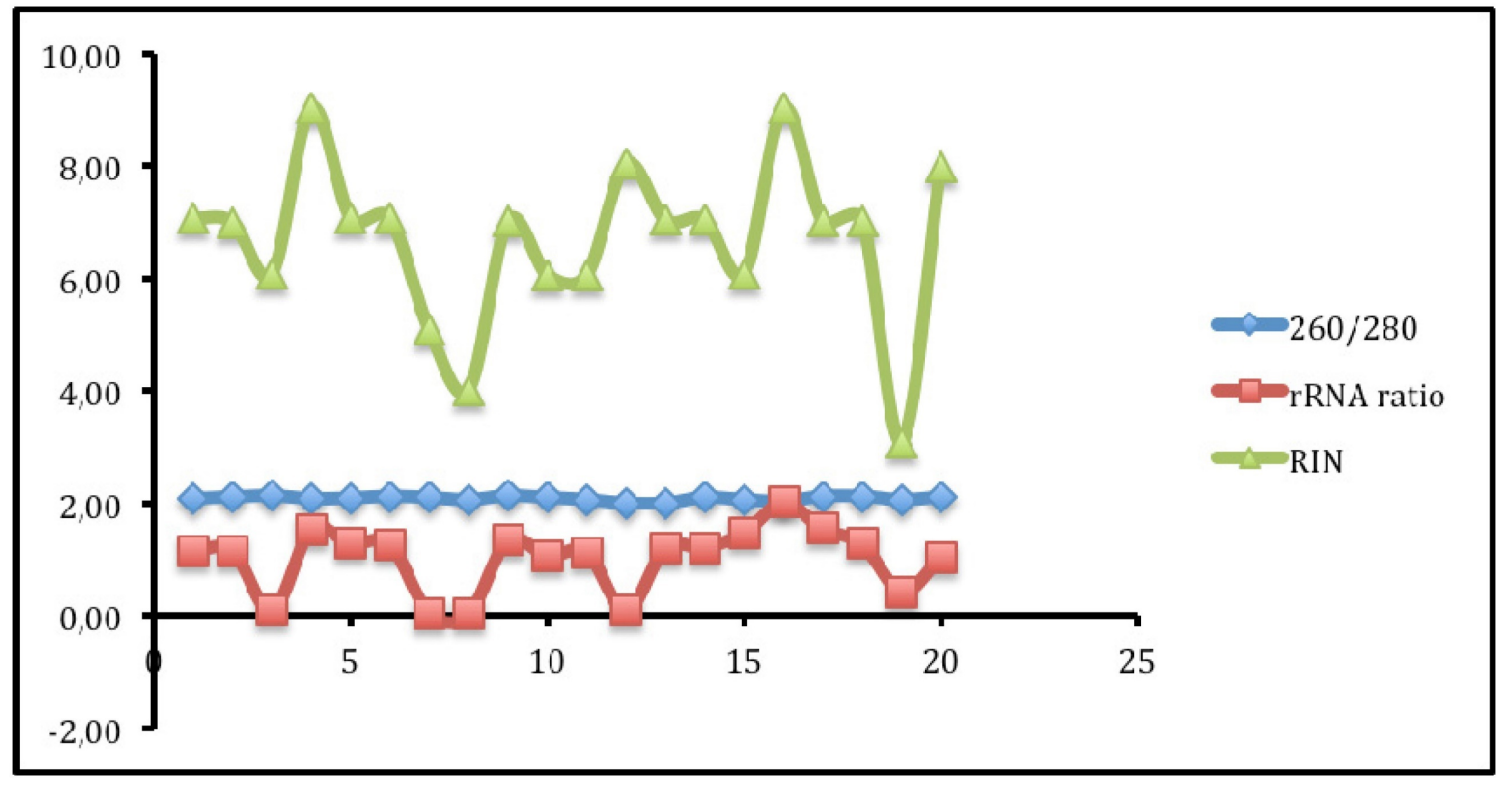
Correlations between the OD, rRNA and RIN show that no matter the level of OD, RIN and rRNA values are not affected.

Figure 2 shows that no matter the level of OD, RIN and rRNA values are not affected. These values are therefre very important values used to determine quality of RNA in differernt stages in genomics. Each test measures particular aspects of the nucleotide that may not necessary affect the following down stream test. Therefore, all these QC tests are very useful in the RNA-seq process.

### Further quality control of sequenced data

Quality control continues even beyond the level of nucleotides. When data is sequenced, it can be used to verify if the samples were degraded or if there have been any mix up in the samples.

### Hierarchical Clustering

**Figure 3:**
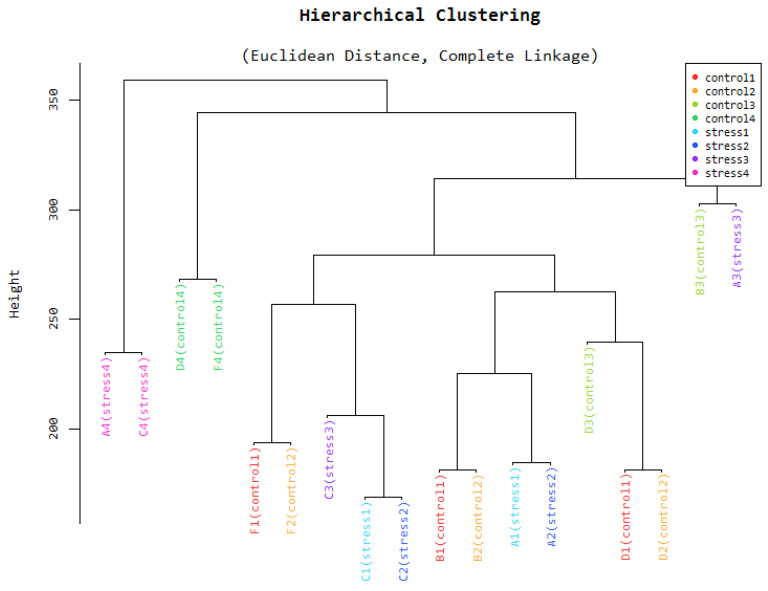
Hierarchical clustering.

Figure 3 is a hierarchical clustering obtained from the different samples used in the study. The samples are tissues obtained in different organs of the plant. These samples also have different physiological states. The Euclidean distances between samples enable us to check the conformity and quality of our samples. According to the figure, samples that were taken from adjacent tissues tend to be very close together while those collected further apart tend to have a larger Euclidean distance. This representation gives the researcher the assurance that the final results of downstream applications can be relied upon as excellent. RIN values are given a very important consideration when sequencing is being done.

This is an additional method that can be used to determine the level of similarity of our samples. The figure shows that all samples collected in adjacent tissues are closely related and fall in the same principal component. This will prove to the researchers if their samples have not been adulterated in the course of the study. Multidimensional scaling is a proximity matrix of the different pairwise data sets.

### Significant transcript count between the organs

We recall that RNA samples were collected on 4 different parts of the oil palm. The stages 1, 2, 3, 4 are represented in the figure as stress 1 or stress 2 and so on. The stages are numbered in ascending values of distance between them. Stage No. 1 is closer to stage No. 2 and further from stage No. 3 and No. 4.

We can clearly observe that there are only 293 significant transcripts that are differentially expressed between stress 1 and stress 2. Stress 1 tissues are taken from the very early stage of flowering in oil palm as described in 15. Stress 2 is collected from young flower tissues next to stress 1. This is not the case in other comparisons such as between Stress 4 and stress 1. Stress 4 are tender leaf tissues while stress 1 are very early flower bud tissues. Therefore, the differentially expressed genes between these relatively distant tissues are very large, giving a total of 2566 DEGs. This is an additional reverse verification of the quality of nucleotides that have been used in a scientific study. Similarly, we observe that all comparisons with the leaf tissue against all 3 stages of flower tissue have larger amounts of significant DEGs. Therefore the further the distance between any two physiological stages, the more the number of DEGs between them.

## Conclusions

RIN and other QC measurements have stood out independent of OD measured in field labs. Differences in OD values didn't distort the truthfulness of the samples in down stream quality control measurements. Therefore, RNA samples should not be discarded in the field labs because of poor OD results. These values are separate values that must be taken into consideration, each at a particular stage of RNA quality control. Post sequencing quality control measures can be used implicitly to prove that the original samples went throughout the process without any interference or adulteration.

## Figures and Tables

**Figure 4:**
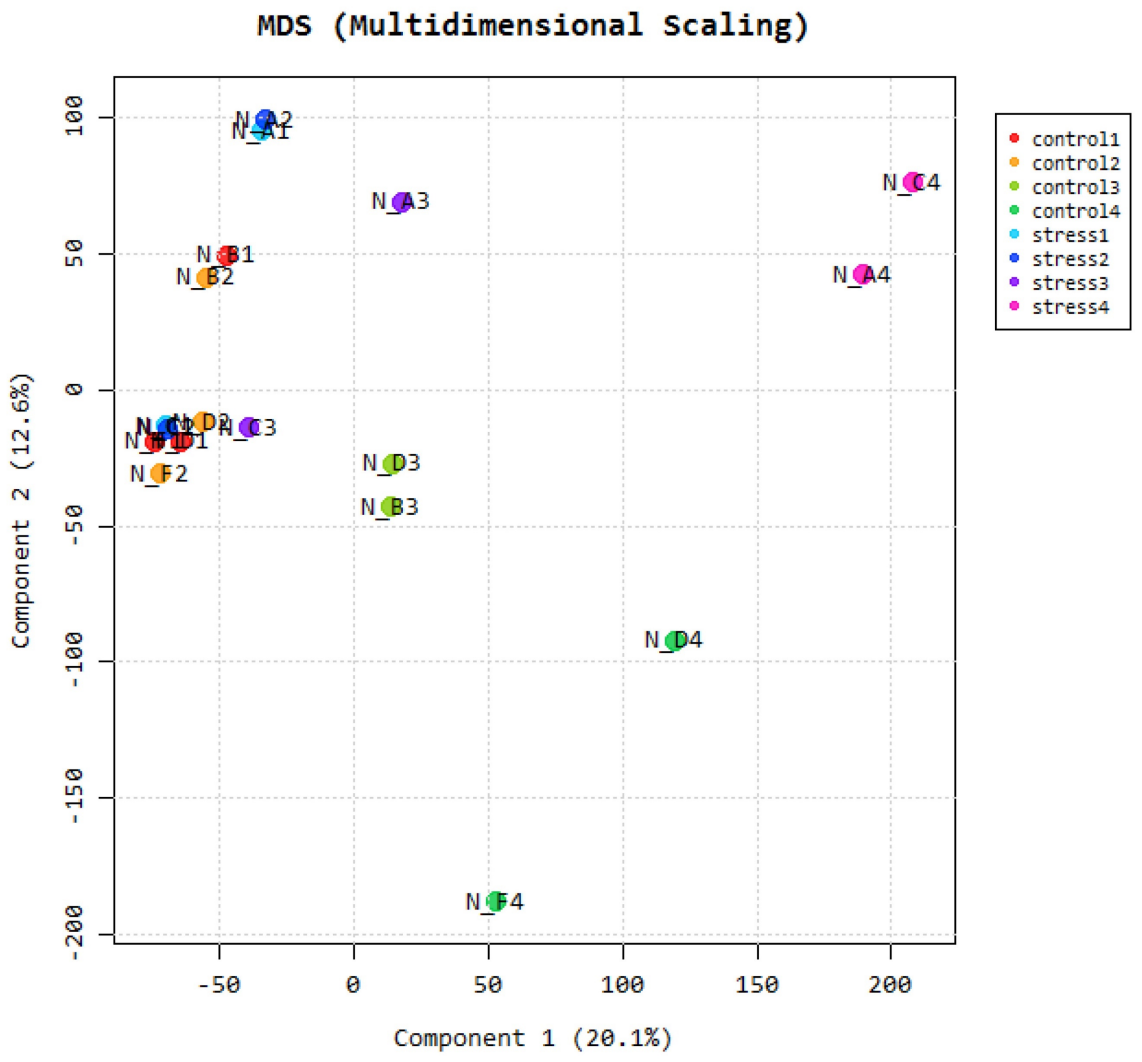
Multidimensional scaling.

**Figure 5:**
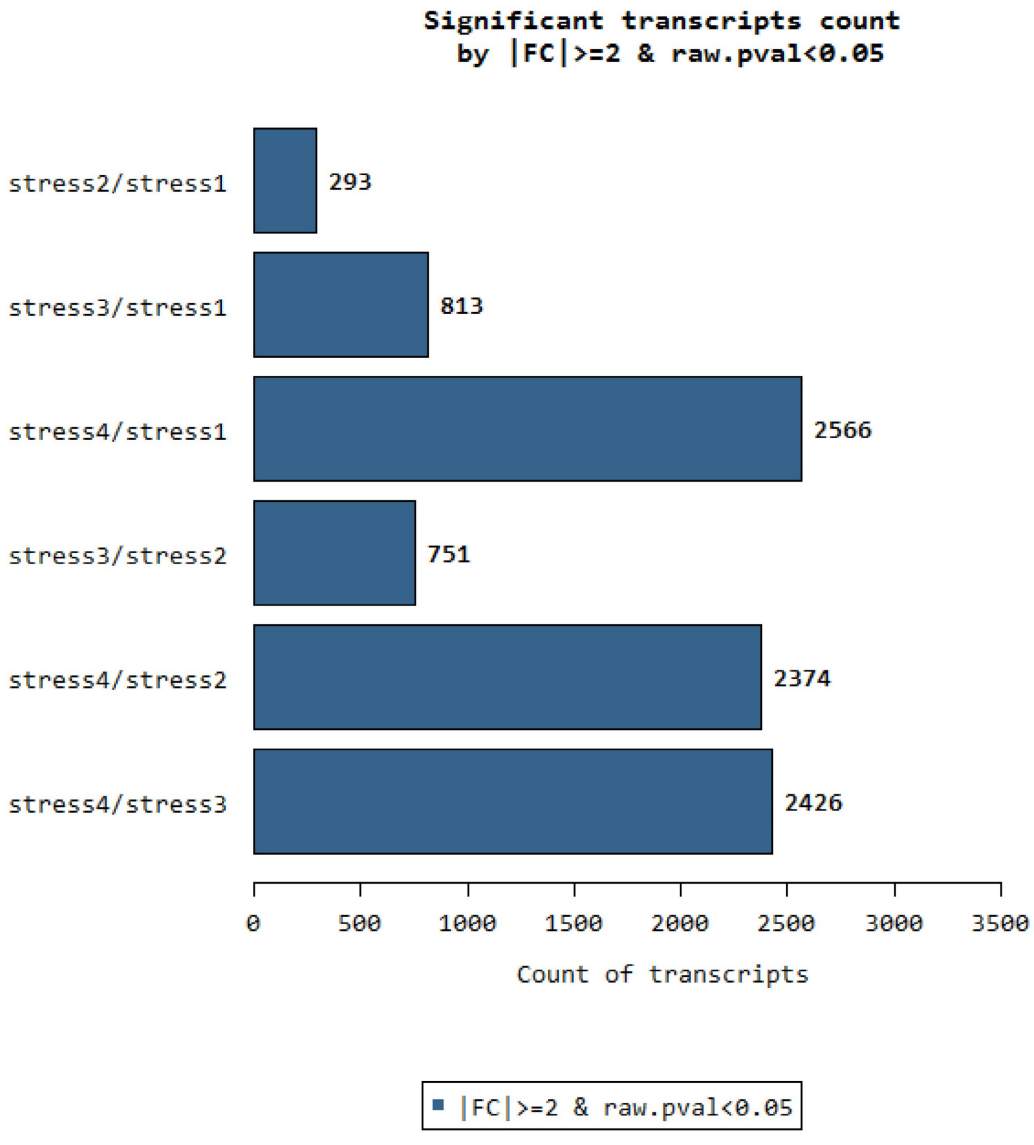
Significant transcript counts between comparing tissues.

**Table 1:** OD for different RNA samples

Code	Conc.	Unit	A260	A280	260/280	260/230
A1	392,7	ng/µl	7,854	3,774	2,08	1,79
A2	980,6	ng/µl	19,613	9,282	2,11	1,54
A3	565,5	ng/µl	14,137	6,636	2,13	1,61
A4	866,8	ng/µl	21,669	10,425	2,08	1,71
B1	1291	ng/µl	32,276	15,445	2,09	2,26
B2	1083,1	ng/µl	27,078	12,783	2,12	2,27
B3	229,7	ng/µl	5,741	2,733	2,1	1,51
B4	371,4	ng/µl	9,286	4,498	2,06	1,24
C1	1345,3	ng/µl	26,906	12,624	2,13	2,04
C2	1621,4	ng/µl	32,427	15,448	2,1	2,25
C3	570,4	ng/µl	11,408	5,54	2,06	2,21
C4	389	ng/µl	7,779	3,887	2	1,31
D1	754	ng/µl	15,08	7,539	2	1,44
D2	1123,2	ng/µl	22,463	10,722	2,1	2,33
D3	646,3	ng/µl	12,926	6,284	2,06	2,32
D4	104,2	ng/µl	2,084	1,027	2,03	1,98
F1	1411,7	ng/µl	35,292	16,661	2,12	2,27
F2	926,4	ng/µl	23,161	10,925	2,12	2,31
F3	419,4	ng/µl	10,485	5,082	2,06	2,26
F4	319,8	ng/µl	7,995	3,792	2,11	1,98

**Table 2:** RIN Values for different samples

Sample Name	Conc, (ng/ul)	RIN	rRNA ratio
A1	122.367	7.7	1.16
A2	616.038	7	1.06
A3	233.546	6.8	0.9
A4	227.137	9.4	1.53
B1	495.388	7.8	1,29
B2	463.323	7.7	1.27
B3	505.016	5.8	0.77
B4	294.544	4.2	0.3
C1	551.086	7.2	1.35
C2	556.761	6.6	1.07
C3	602.935	6.6	1.13
C4	193.945	8.4	1.83
D1	252.018	7.4	1.21
D2	390.933	7.4	1.22
D3	209.945	6.9	1.48
D4	104.924	9.3	2.2
F1	842.423	7.9	1.55
F2	465.768	7.3	1.28
F3	275.642	3.9	0.43
F4	238.273	8	1.4
